# A Decline in Follicle Cell Function Is a Major Driver of *Drosophila* Ovarian Aging

**DOI:** 10.1111/acel.70529

**Published:** 2026-04-30

**Authors:** Emily A. Wolfgram, Todd G. Nystul

**Affiliations:** ^1^ Department of Anatomy UCSF San Francisco California USA; ^2^ Bakar Aging Research Institute UCSF San Francisco California USA; ^3^ Broad Center of Regeneration Medicine and Stem Cell Research UCSF San Francisco California USA; ^4^ Department of OB‐GYN/RS UCSF San Francisco California USA

**Keywords:** aging, cell biology, *drosophila*, ovary, reproduction

## Abstract

The ovary is one of the first organs to lose functionality with age. We found that aging of the *Drosophila* ovary is characterized by an accumulation of phenotypes in the somatic compartment, including failure of the follicle cells to encapsulate germ‐cell cysts, an extended S phase, and increased DNA damage. In aged ovaries, follicle encapsulation defects are associated with the lack of a germ‐cell cyst checkpoint in early oogenesis. Single‐cell RNA sequencing revealed that, across all cell types in the ovary, cells in the follicle lineage have the highest number of differentially expressed genes. Overexpression of *Atg8a*, a key autophagy machinery gene homologous to mammalian LC3, specifically in follicle cells prevents age‐associated decline in the follicle epithelium and loss of reproductive capacity. Collectively, these findings demonstrate that genetic manipulation of a small population of ovarian somatic cells is sufficient to improve both cell‐autonomous and non‐autonomous features of reproductive aging.

## Graphical abstract

Aging drives a progressive accumulation of defects in somatic follicle cells of the *Drosophila* ovary. These defects are accompanied by reduced tissue and genome integrity, altered transcriptional profiles, and disrupted germ‐soma coordination. Targeted overexpression of *Atg8a* in a subset of follicle cells mitigates these defects and preserves reproductive capacity with age.

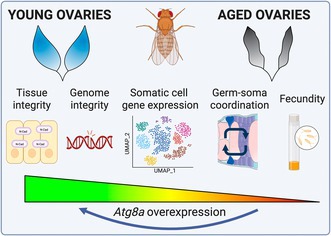

## 1 Introduction

Aging is an inevitable biological process that leads to the decline of cellular functionality (López‐Otín et al. 2013). Among the organs affected, the ovary is one of the first to lose function, with females reaching menopause at an average age of 51 (Desai and Rajkovic 2017). While ovarian morphology varies between species, the mechanisms governing oogenesis are highly conserved. From *Drosophila* to higher‐order mammals, oogenesis involves the progression of early germ cells into fully developed eggs (Spradling et al. 2022). There are broad similarities in organ morphology, with many direct parallels between cell types and stages of oogenesis, as well as several conserved features at the molecular and cellular level. Both *Drosophila* and mammalian ovaries have germ cells that undergo multiple rounds of incomplete division, forming multicellular cysts (Fellmeth and McKim 2022). In the somatic compartment, the follicle cells in the *Drosophila* ovary functionally parallel the granulosa cells in the mammalian ovary. Both follicle cells and granulosa cells directly contact germ‐cell cysts and proliferate during oogenesis to maintain a single continuous layer that forms the surface of the growing follicle (Doherty et al. 2022). In both fly and mammalian ovaries, these cells help guide the process of oogenesis via direct cell–cell communication with germ cells through gap junctions (Stebbings et al. 2002; Carabatsos et al. 2000) and the production of endocrine signals that coordinate the progression of germ cell development with the actions of other cell types (Belles and Piulachs 2014; Krajnik et al. 2023).

The *Drosophila* ovary is comprised of ~16 strands of ovarioles, with a germarium at the anterior end of each ovariole. The germarium is where oogenesis begins and is host to both the germline stem cells (GSCs) and follicle stem cells (FSCs). At the anterior tip of the germarium reside the GSCs, which asymmetrically divide to self‐renew and produce a germ cell. Each germ cell undergoes four rounds of incomplete mitosis, producing a 16‐cell germline cyst that moves posteriorly into the germarium at a position called the Region 2a/2b border. At this position in the germarium, a population of follicle stem cells (FSCs), supported by an escort cell (EC) niche, asymmetrically divide to self‐renew and produce pre‐follicle cells (pFCs) (Rust and Nystul 2020; Sahai‐Hernandez et al. 2012). These pFCs will continue to divide and differentiate into polar cells, stalk cells, and main body (MB) follicle cells. When a germline cyst reaches region 3 of the germarium, it buds off, encapsulated by MB follicle cells, and is connected to other encapsulated cysts via stalk cells. Follicle cells continue to divide and grow to maintain encapsulation of the developing cyst, prior to full egg development (King et al. 1956). Given the continuous demand for follicle cells to encapsulate and guide the germline cyst through oogenesis, FSCs and pFCs are among the most proliferative cells in adult *Drosophila* (Margolis and Spradling 1995). This makes the FSC lineage particularly susceptible to replicative stress and may make it more vulnerable to other insults that are known to increase with age including stem cell maintenance, reactive oxygen species production, mitochondrial dynamics, and autophagy regulation (López‐Otín et al. 2013; Balough et al. 2024; Pan et al. 2007; Boyle et al. 2007; Borch Jensen et al. 2017; Nagy et al. 2018). Any stressors that impair FSC proliferation and differentiation could have severe consequences for fertility. Indeed, genetic manipulations that disrupt FSC activity often result in significant morphological defects and, in some cases, sterility. These findings underscore the crucial role of the follicle lineage in *Drosophila* ovarian aging.

The average lifespan of wildtype *Drosophila* is approximately 70 days (Piper and Partridge 2018), yet fertility begins to decrease after the first week of adulthood and drops below 10% of the maximum rate by 50 days (Novoseltsev et al. 2002). The causes of this decrease in fertility are not fully understood but, in Drosophila, as in mammals, it involves a decline in germ cell number and germ cell‐niche interactions, which are strongly influenced by nutrition and stress signaling pathways, along with marked changes in the transcriptional profile (Jones 2007; Grandison et al. 2009; Pan et al. 2007; Boyle et al. 2007; Even‐Ros et al. 2025; Ishibashi et al. 2020; Kao et al. 2015; Ameku et al. 2018; Zhao et al. 2008; Lu et al. 2023; Jin et al. 2025; Gaylord et al. 2025). Likewise, emerging evidence from studies of both *Drosophila* and mammals is beginning to elucidate the important role of ovarian somatic cell aging in the loss of fertility (Singh et al. 2018; Gaylord et al. 2025; Wang et al. 2024; Lu et al. 2023; Jin et al. 2025).

In this study, we conducted a phenotypic analysis of aged ovaries and identified cellular and tissue‐scale aberrations in the typically well‐organized layer of follicle cells. In addition, we performed single‐cell RNA sequencing and found a particularly high number of transcriptional differences in the FSC lineage between young and aged ovarioles. We identified *Atg8a*, an upstream effector in the autophagy pathway, as a key regulator of follicle cell aging and reproductive capacity. These results highlight a new avenue for extending reproductive longevity through manipulation of the somatic lineage rather than the germline, which is ultimately passed on to offspring.

## 2 Methods

### 2.1 Fly Husbandry and Aging Time Course

Flies were maintained on standard molasses food at 25°C. The following stocks from Bloomington Drosophila Stock Center (BDSC) were used in this study: *w1118(x);;* (3605);*109‐30‐Gal4;* (7032), and;*UAS*‐*Atg8a::mCherry‐GFP;* (37749).*;;Lamp1::3x‐mCherry* was described in Hegedűs et al. (2016). We used FlyBase to find information on phenotypes/function/stocks/gene expression. For all aging assays, virgin females were collected and paired with males and maintained in standard lab conditions until dissections at weekly timepoints. Age was calculated as weeks post‐eclosion.

### 2.2 Immunofluorescence Staining and Imaging

*Drosophila* ovaries were dissected in Phosphate‐Buffered Saline (PBS) and ovaries fixed in 4% Paraformaldehyde (PFA) + PBS for 15 min. Ovaries were rinsed with PBS before blocking for 1 h in standard block (PBS + 0.2% Triton X‐100 + 0.5% bovine serum albumin). Ovaries were incubated in primary antibodies with block overnight on a nutator at 4°C in dark conditions. The following day, ovaries were rinsed with block for 1 h before adding secondary antibodies. Ovaries were incubated in secondary antibodies with block overnight on a nutator at 4°C. The following day, ovaries were rinsed three times with PBS for 5 min each. Once the PBS was removed, DAPI‐Fluoromount G Slide Mounting Media (SouthernBioTech) was added, and ovaries were stored at 4°C in dark conditions until mounted on slides. Ovaries were mounted on glass slides in DAPI‐Fluoromount G Slide Mounting Media. Primary antibodies used were: mouse anti‐Fas3 7G10 (1:100, Developmental Studies Hybridoma Bank (DSHB)), rat anti‐Vasa 46F11 (1:200, DSHB), rat anti‐CadN DN‐Ex#8 (1:100, DSHB), rabbit anti‐pHH3 (Ser10, 1:500, Millipore Sigma), mouse anti‐UNC93‐5.2.1 (gH2Av, 1:100, DSHB), and rabbit anti‐Vkg (1:1000, Dr. Stephane Noselli), rat anti‐RFP 5F8 (1:500, Chromatek), mouse anti‐Rab7 (1:100, DSHB). The following secondary antibodies were used at 1:500: goat anti‐mouse 488 (Life Technologies, A11001), goat anti‐rabbit 488 (Invitrogen, A11034), goat anti‐rat 488 (Invitrogen, A11006), goat anti‐mouse 555 (Invitrogen, A21422), goat anti‐rat 555 (Invitrogen, A21434), goat anti‐mouse 647 (Invitrogen, A21236), goat anti‐rat 647 (Invitrogen, A21247).

Images were acquired with a Zeiss M2 Axioimager with an Apotome unit using Zen 3.5 software. Initial image processing was performed in Zen using the Apotome Raw Convert feature. All further image processing was done in FIJI, and figures were prepared in Adobe Illustrator.

### 2.3 Image Analysis

Analyses for N‐cadherin prevalence, pHH3, EdU, gH2Av, TUNEL, and Lysotracker were performed by hand‐counting. Vkg intensity was performed by outlining the germarium with a segmented line and taking the intensity of Vkg signal after background subtraction.

### 2.4 EdU Staining

For EdU experiments, ovaries were dissected in PBS and incubated in 0.75uL EdU Click‐IT Alexa Fluor 555 Imaging Kit (ThermoFisher Scientific, C10338) with 499.25uL PBS at room temperature for 1 h. Ovaries were then fixed in 4% PFA + PBS for 10 min and then rinsed in PBS prior to blocking for 1 h in PBS + 0.2% Triton X‐100 + 0.5% bovine serum albumin. Primary antibodies were then added, and ovaries were stored overnight at 4°C on a nutator in dark conditions. The following day, ovaries were rinsed in PBS twice and incubated in a reaction mix (430 μL 1× Click‐IT EdU Reaction Buffer, 20 μL CuSO_4_, 1.2 μL Alexa Fluor 555 azide, 50 μL 10× Click‐IT EdU Buffer Additive in deionized water) for 1 h at room temperature on a nutator in dark conditions. The ovaries were then rinsed twice with PBS and blocked for 1 h. Secondary antibodies were added with block and ovaries were stored overnight at 4°C on a nutator in dark conditions. Ovaries were then labeled with secondary antibodies according to the standard Immunofluorescence Staining protocol described above.

### 2.5 LysoTracker Staining

For LysoTracker experiments, ovaries were prepared according to the immunofluorescence staining protocol described above, except that they were incubated in LysoTracker Red DND‐99 (ThermoFisher Scientific) at a 1:100 dilution in PBS for 5 min prior to fixation, washed with PBS for 5 min, and then fixed in 4% PFA + PBS for 10 min rather than the standard 15 min. Ovaries were then labeled with primary and secondary antibodies according to the standard immunofluorescence staining protocol described above.

### 2.6 TUNEL Staining

For TUNEL experiments, the following protocol was adapted from the Click‐iT TUNEL Alexa Fluor Imaging Assays 488 kit (ThermoFisher Scientific, C10617). Ovaries were dissected in PBS and fixed in 4% PFA + PBS for 10 min and then rinsed in PBS. Cell membranes were permeabilized in PBS + 0.2% Triton X‐100 for 15 min. Ovaries were rinsed twice with PBS and then primed with TdT Reaction Buffer for 10 min. Ovaries were incubated at 37°C for 1 h in a TdT reaction cocktail (47 μL TdT Reaction Buffer + 1 μL EdUTP, and 2 μL TdT enzyme). A reaction supermix was prepared and stored at 4°C (2630 μL 1× Click‐iT Plus TUNEL Reaction Buffer, 67 μL copper protectant, and 3.7 μL 488 picolyl azide). Ovaries were rinsed twice with PBS and incubated at 37°C for 30 min in a Click‐iT reaction cocktail (45 μL reaction supermix and 5 μL 10× Click‐iT Plus TUNEL Reaction Buffer Additive), protected from light. Ovaries were then labeled with primary and secondary antibodies according to the standard Immunofluorescence Staining protocol described above.

### 2.7 Egg Laying Assay

For egg counts, flies were separated into vials containing two females and one male for each genotype, on standard food at 25°C. After 24 h, eggs were hand counted through a dissection microscope and recorded. Total egg counts were divided by 2, so that final data represents the number of eggs per female in a 24‐h period.

### 2.8 Single‐Cell RNA Sequencing Prep

Preparation for single‐cell RNA sequencing of *w1118(x);;* ovaries at 1 and 6 weeks old followed the protocol as described in Meyer et al. (2023). However, we did not enrich for the anterior region, as this caused the too much cell loss in the aged condition. The single‐cell RNA sequencing was performed at UCSF CoLabs using the 10X Genomics Single Cell 3′ kits (v3.3 and v4). Sequencing was performed on Illumina NovaSeq X at the UCSF Center for Advanced Technology (CAT).

### 2.9 Bioinformatic Analysis

Sequencing reads were aligned to the *Drosophila* reference genome using the 10X Genomics Cloud CLI, which performs Cell Ranger alignment and initial quality filtering. The standard Seurat pipeline (v5) was applied to each individual dataset to filter out low quality cells based on the number of RNA features, number of RNA counts, and percent mitochondria reads (Figure S4A–D). Each dataset was individually normalized and scaled. Anchors from the 1‐week dataset were applied to the 6‐week dataset. Principal components and clustering neighbor parameters were determined by the strength of the silhouette scores and mean distance within a cluster, as well as identification of known transcript expression for clusters. This workflow resulted in a single Seurat object with 20,818 high‐quality cells, grouped into 9 clusters, identified by known transcripts. We used FlyBase (including release FB2026_01 and prior releases) to find information on phenotypes, gene functions, and stocks (Öztürk‐Çolak et al. 2024).

### 2.10 Statistics and Data Availability

All analyses for single‐cell RNA sequencing (Figure 4, Figure S3, Tables S1–, S3) were performed in R. FASTQ are available on GEO (accession # GSE318563). All other statistical analyses were performed in R and graph generation was performed in Python. Boxplots show the median and interquartile values, and whiskers show the range. Barplots show the mean and error bars show the standard deviation. All quantification is a result of at least three independent replicates. A Levene test was used to test for equal variance in cases where a Student's *t*‐test was used. Raw data files, code for generating graphs and statistical analyses, and code for building the Seurat object are available via the GitHub repository (https://github.com/NystulLab/WolfgramAgingOvary.git).

## 3 Results

### 3.1 Establishment of Follicle Cell Phenotypes Associated With Aging in the *Drosophila* Ovary

**FIGURE 1:**
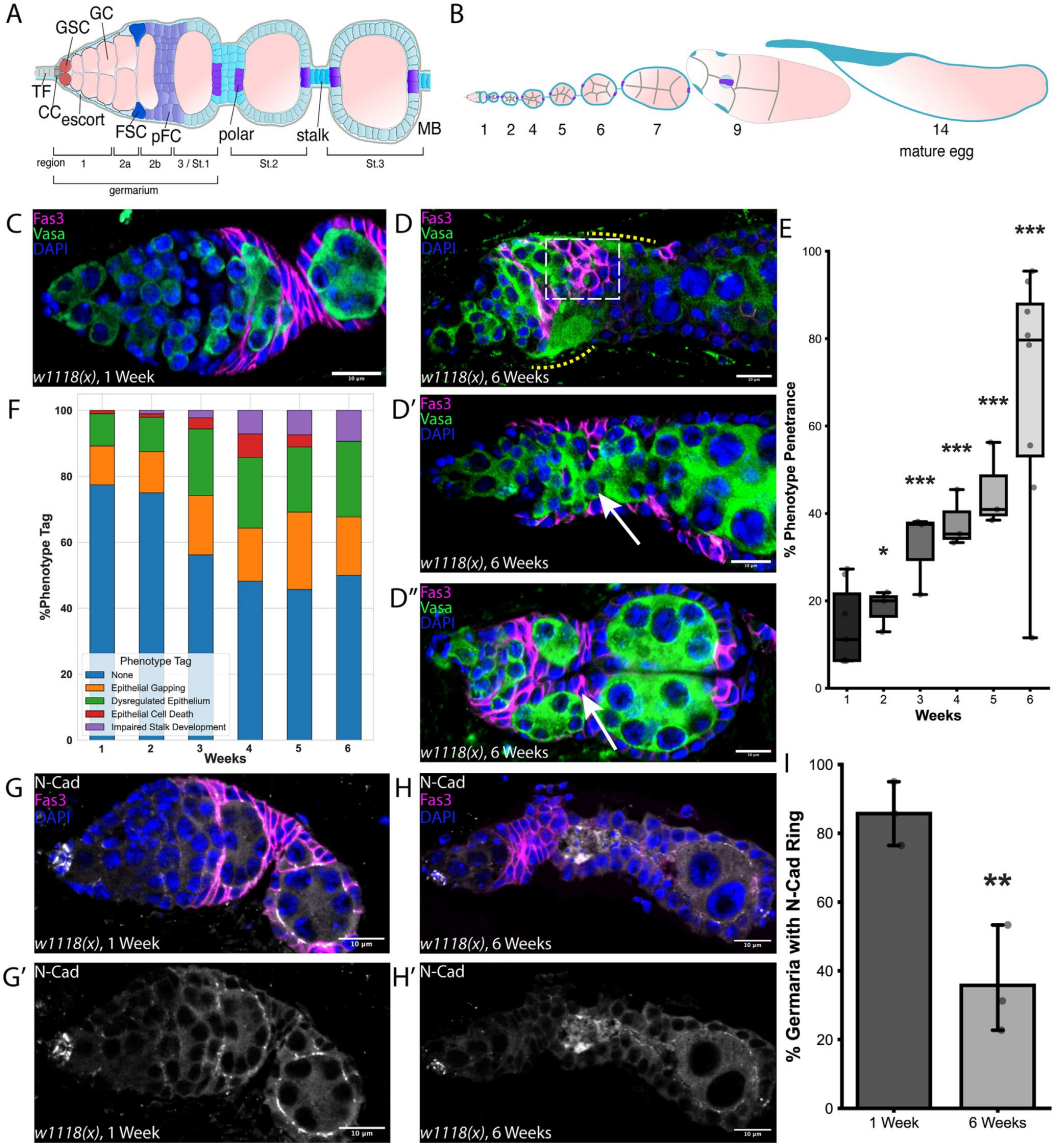
Aging leads to increased follicle cell phenotypes in the *Drosophila* ovary. (A) Schematic of the *Drosophila* germarium, positioned at the anterior tip of the ovariole. TF: terminal filament, CC: cap cell, GSC: germline stem cell, GC: germ‐cell cyst, FSC: follicle stem cell, pFC: prefollicle cell, MB: main body, St: Stage. (B) Schematic of the ovariole. (C) Germarium from a *w11118(x);;* fly at 1 week old, stained for Fas3 (magenta), Vasa (green), and DAPI (blue). (D–D″) Representative examples of germaria from w1118(x);; flies at 6 weeks old. Yellow dotted lines show epithelial gapping, white dashed box shows epithelial dysregulation and epithelial cell death, white arrows show impaired stalk development. (E) Quantification of the percent of germaria with follicle cell phenotypes at weekly timepoints from 1 to 6 weeks old. *n* = 298, 88, 73, 43, 64, and 201 germaria, respectively. (F) Quantification of follicle cell phenotype tags from 1 to 6 weeks old. *n* = 98, 88, 73, 43, 64, and 77 germaria, respectively. (G) Germarium from a *w11118(x);;* fly at 1 week old, stained for N‐cad (gray), Fas3 (magenta), and DAPI (blue). (G′) N‐cadherin ring forms around developing germ‐cell cysts in 1‐week‐old germaria. (H) Germarium from a *w11118(x);;* fly at 6 weeks old, stained for N‐cad (gray), Fas3 (magenta), and DAPI (blue). (H′) Loss of N‐cadherin ring around developing germ‐cell cysts in a 6‐week‐old germarium. (I) Quantification of the percent of germaria with the N‐cad ring around germ‐cell cysts at 1 and 6 weeks old. *n* = 58 and 53 germaria, respectively. Scale bars are 10 μm. ns = not significant, *p < 0.05, **p < 0.01, ***p < 0.001, using Dunnett's Test (E) and Student's *t*‐test (I).

To establish hallmarks of follicle cell aging, we assayed for phenotypes in the follicle epithelium at weekly intervals from 1 to 6 weeks post eclosion in ovarioles from *w1118(x)* flies. In healthy ovarioles, follicles bud from the germarium in a single file, are surrounded by an intact, single‐layered follicle epithelium, and are separated from one another by a single row of stalk cells (Figure 1A,C). Ovarioles with follicles that deviated from this pattern were scored as having a phenotype. The frequency of ovarioles with these phenotypes progressively increased over the 6‐week time course (Figure 1D,E). We therefore designated the 1‐week‐old ovary as the “young” condition and 6‐week‐old ovary as the “aged” condition. Qualitative assessment of these follicle cell phenotypes produced four categories (Figure 1F, Figure S1A–D). The two most prominent categories were epithelial gapping, where follicle cells, identified by the marker Fasciclin3 (Fas3), are absent from the perimeter of the germarium or developing follicle (Figure 1D, yellow short‐dashed line), and epithelial dysregulation, in which the follicle cells were misshapen or failed to form into a single‐layered epithelium (Figure 1D, white long‐dashed box). We also observed Fas3^+^ cells with a rounded morphology consistent with epithelial cell death (Figure 1D, white long‐dashed box), and a small number of instances of impaired stalk development (Figure 1D″–D″′, white arrows).

The loss of follicle cell integrity in aged germaria prompted us to investigate whether another follicle cell adhesion protein, N‐cadherin (N‐cad) is also diminished with age. N‐cad is highly expressed in prefollicle cells and main body follicle cells from Stage 1 to approximately Stage 5 (Rust and Nystul 2020). N‐cad mediates cell adhesion through homotypic interactions at the apical‐lateral surface of the cells (Yonekura et al. 2006), creating a characteristic “ring” of N‐cad signal between follicle cells and germ cells (Figure 1G,G′). We found that this N‐cad ring is frequently lost in aged germaria (Figure 1H,H′,I). In contrast, we found no significant difference between young and aged germaria in the level of Collagen IV (*Vkg*) in the basement membrane (Figure S2A–C,B′,C′). Likewise, we performed a TUNEL assay to identify apoptotic cells and found that the frequency does not significantly change in aged conditions (Figure S2C–E,C′–D′, yellow arrows) (Crowley et al. 2016). However, the overall low rates of apoptosis in this tissue may make it difficult to detect changes. Taken together, these data establish quantitative and qualitative hallmarks of follicle cell aging in the *Drosophila* ovary.

### 3.2 Characterization of Cell Cycle Regulation in Aging Follicle Cells

**FIGURE 2:**
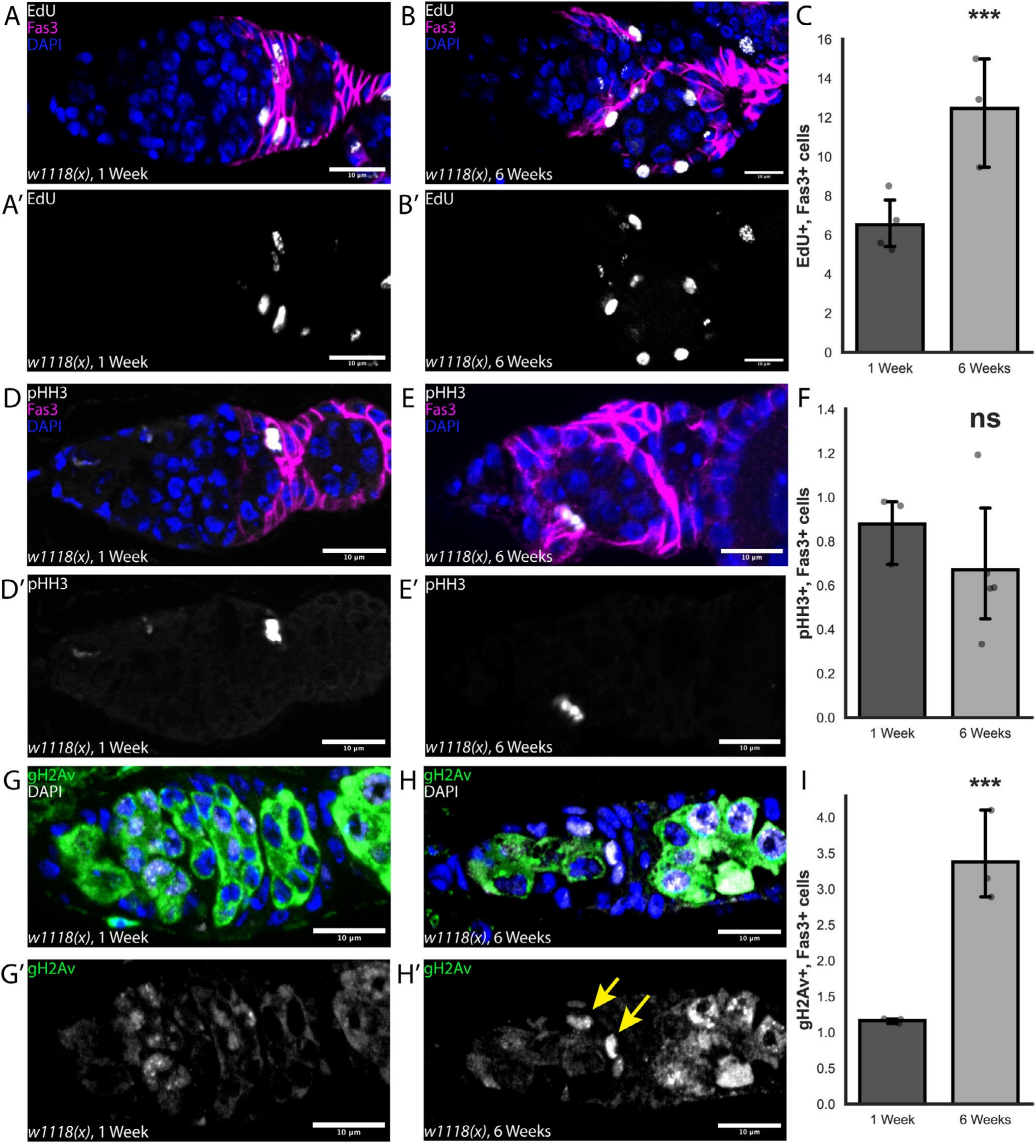
Aging leads to an extended S‐phase and increased DNA damage in follicle cells. (A, B) Germarium from *w1118(x);;* flies at 1 (A) or 6 (B) weeks old, stained for EdU (gray), Fas3 (magenta), and DAPI (blue). (A′, B′) EdU identifies cells that are in S‐phase during the 1‐h incubation prior to fixation. (C) Quantification of the number of EdU+, Fas3+ cells per germarium at 1 and 6 weeks old. *n* = 98 and 65 germaria, respectively. (D, E) Germarium from *w1118(x);;* flies at 1 (D) or 6 (E) weeks old, stained for pHH3 (gray), Fas3 (magenta), and DAPI (blue). (D′, E′) pHH3 identifies cells that are in mitosis. (F) Quantification of the number of pHH3+, Fas3+ cells per germarium at 1 and 6 weeks old. *n* = 200 and 124 germaria, respectively. (G, H) Germarium from *w1118(x);;* flies at 1 (G) or 6 (H) weeks old, stained for gH2Av (gray), Fas3 (magenta), and DAPI (blue). (G′, H′) gH2Av identifies sites of DNA damage. (I) Quantification of the number of highly positive gH2Av+, Fas3+ cells per germarium at 1 and 6 weeks old. *n* = 35 and 39 germaria, respectively. Scale bars are 10 μm. ns = not significant and ***p < 0.001, using Poisson Dispersion Test (C, F, I).

The FSC lineage is highly proliferative, undergoing up to nine rounds of division before exiting the cell cycle at Stage 6 (Margolis and Spradling 1995). We therefore investigated whether the cell cycle of follicle cells is altered with age. First, we used an EdU assay to quantify the number of follicle cells per germarium in S‐phase in ovarioles from young and aged flies (Figure 2A,A′,B,B′). Indeed, we observed a statistically significant increase in EdU^+^ cells, from 6.5 ± 1.4 cells per germarium in young flies versus 12.5 ± 2.8 cells per germarium in aged flies (Figure 2C). In contrast, we found no significant difference in the number of follicle cells that were positive for phospho‐histone H3 (pHH3), which is a marker of mitosis (Figure 2D–F,D′–E′). Together, these results indicate that follicle cells in aged ovarioles are spending a greater fraction of time in S‐phase but are not dividing more frequently. We hypothesized that this could be the result of follicle cells in aged germaria requiring more time to accommodate replication stress and DNA damage. Consistent with this, we found that the number of follicle cells per germarium that are positive for the DNA damage marker, gH2Av, significantly increased with age from 1.7 ± 0.03 cells per germarium to 3.4 ± 0.6 cells per germarium (Figure 2G–I).

### 3.3 Follicle Cells Are Unable to Accommodate Germ‐Cell Cysts in Aged Germaria

**FIGURE 3:**
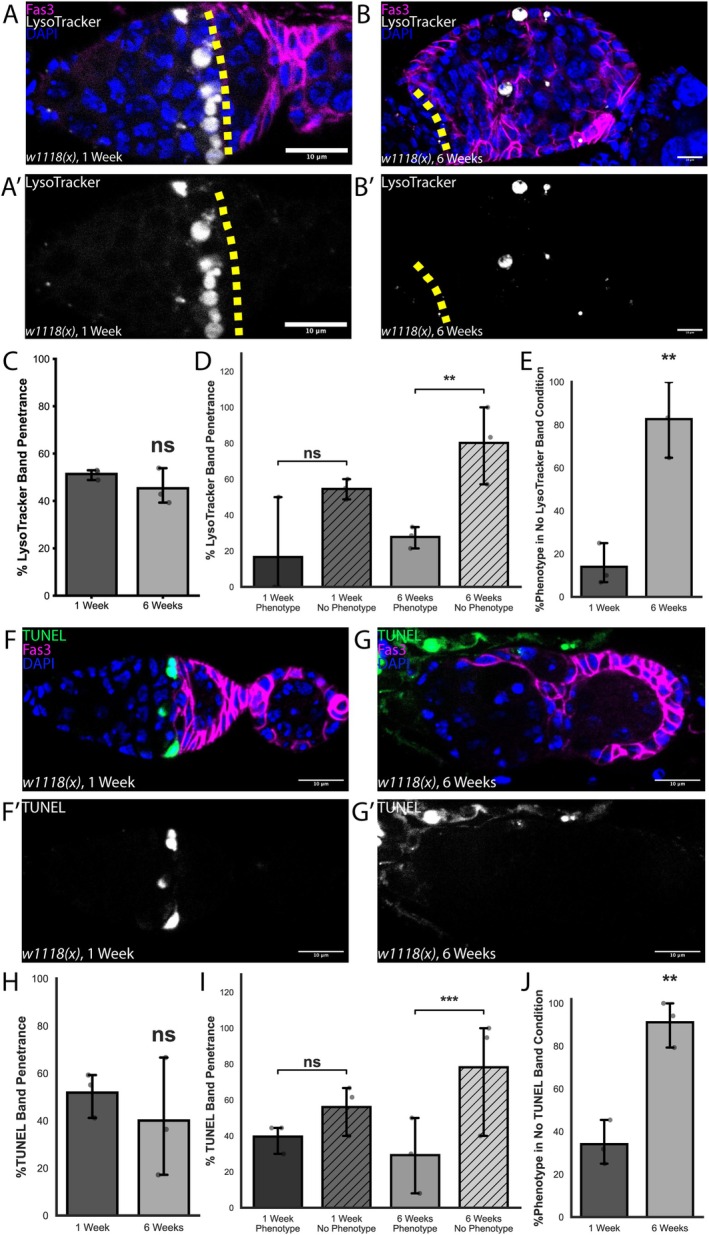
Follicle cell phenotypes are associated with a lack of cyst death in old ovarioles. (A, B) Germarium from *w1118(x);;* flies at 1 (A) and 6 (B) weeks old, stained for Lysotracker (gray), Fas3 (magenta), and DAPI (blue). (A′) A Lysotracker‐positive band may be present anterior to the Region 2a/2b border (yellow dashed line) in 1‐week‐old germaria. (B′) Lysotracker‐positive bands may be absent anterior to the Region 2a/2b border (yellow dashed line) at 6‐week old, phenotype‐positive germaria. (C) Quantification of the percent of germaria with the Lysotracker‐positive band at 1 and 6 weeks old. *n* = 124 and 83 germaria for 1‐ and 6‐week old. (D) Quantification of the percent of germaria at 6 weeks old with the Lysotracker‐positive band in phenotype‐positive and phenotype‐negative germaria. *n* = 57 and 26 germaria, respectively. (E) Quantification of phenotype penetrance in germaria in which no Lysotracker‐positive band is present at 1 and 6 weeks old. *n* = 62 and 47, respectively. (F, G) Germarium from *w1118(x);;* flies at 1 (A) and 6 (B) weeks old, stained for TUNEL (gray), Fas3 (magenta), and DAPI (blue). (F′) A TUNEL‐positive band is present anterior to the Region 2a/2b border in germaria at 1 week old. (G′) TUNEL‐positive bands may be absent anterior to the Region 2a/2b border in phenotype‐positive germaria at 6 weeks old. (H) Quantification of the percent of germaria with the TUNEL‐positive band at 1 and 6 weeks old. *n* = 110 and 119 germaria, respectively. (I) Quantification of the percent of 6‐week‐old germaria with the TUNEL‐positive band in phenotype‐positive and phenotype‐negative germaria. *n* = 87 and 32 germaria, respectively. (J) Quantification of phenotype penetrance in germaria where no TUNEL‐positive band is present at 1 and 6 weeks old. *n* = 53 and 67 germaria, respectively. Scale bars are 10 μm. ns = not significant, **p < 0.01, ***p < 0.001, using Welch's *t*‐test (C, D, H, J) and Binomial General Linearized Mixed Model (D, I).

As the appearance of severe defects in follicle formation is not associated with a substantial change in follicle cell proliferation in aged ovarioles, we next considered whether these phenotypes are associated with a loss of coordination between germ cells and follicle cells. The rate of germ‐cell cyst progression from Region 2a of the germarium into the follicle epithelium in Region 2b is regulated in part by a nutrition‐associated checkpoint. This checkpoint is detectable as an apoptotic cyst in Region 2a, which occurs in approximately 65% of germaria when flies are maintained on standard growth media (Drummond‐Barbosa and Spradling 2001). The dying cysts can be visualized with Apoptag or TUNEL staining and are likely also the source of LysoTracker^+^ germ cells in Region 2a that were reported separately (Drummond‐Barbosa and Spradling 2001; Nezis et al. 2009; Singh et al. 2018). To investigate this checkpoint as a function of age, we first stained young and aged germaria with LysoTracker (Figure 3A,A′). We found that a band of LysoTracker^+^ germ cells was present in Region 2a in approximately 50% of germaria from young flies maintained under the growth conditions we used in this study. Though LysoTracker staining was incompatible with the Click‐iT TUNEL assay, we found that both methods identify structures with the same frequency, shape, and location (Figure 3F–J). In addition, we found that these structures are much larger than typical lysosomes and they do not contain either the canonical lysosome marker Lamp1 or the late endosome marker Rab7 (Figure S3A,B). Together, these observations indicate that both LysoTracker and TUNEL staining identify apoptotic cysts in Region 2a. Surprisingly, the frequency of germaria with a band of LysoTracker^+^ germ‐cell cysts did not change in the aged condition (Figure 3C), indicating that this checkpoint does not decline with age. However, we found that ovarioles from aged flies without LysoTracker^+^ germ‐cell cysts in Region 2a were significantly more likely to have one or more age‐associated follicle cell phenotypes, whereas no such association was found in ovarioles from young flies (Figure 3B′,D). This suggests that the increase in follicle cell phenotypes with age is linked to a lack of coordination between germ cells and follicle cells.

### 3.4 Single‐Cell RNA Sequencing of Aged *Drosophila* Ovaries Reveals Key Transcriptional Differences in the Follicle Cell Lineage

**FIGURE 4:**
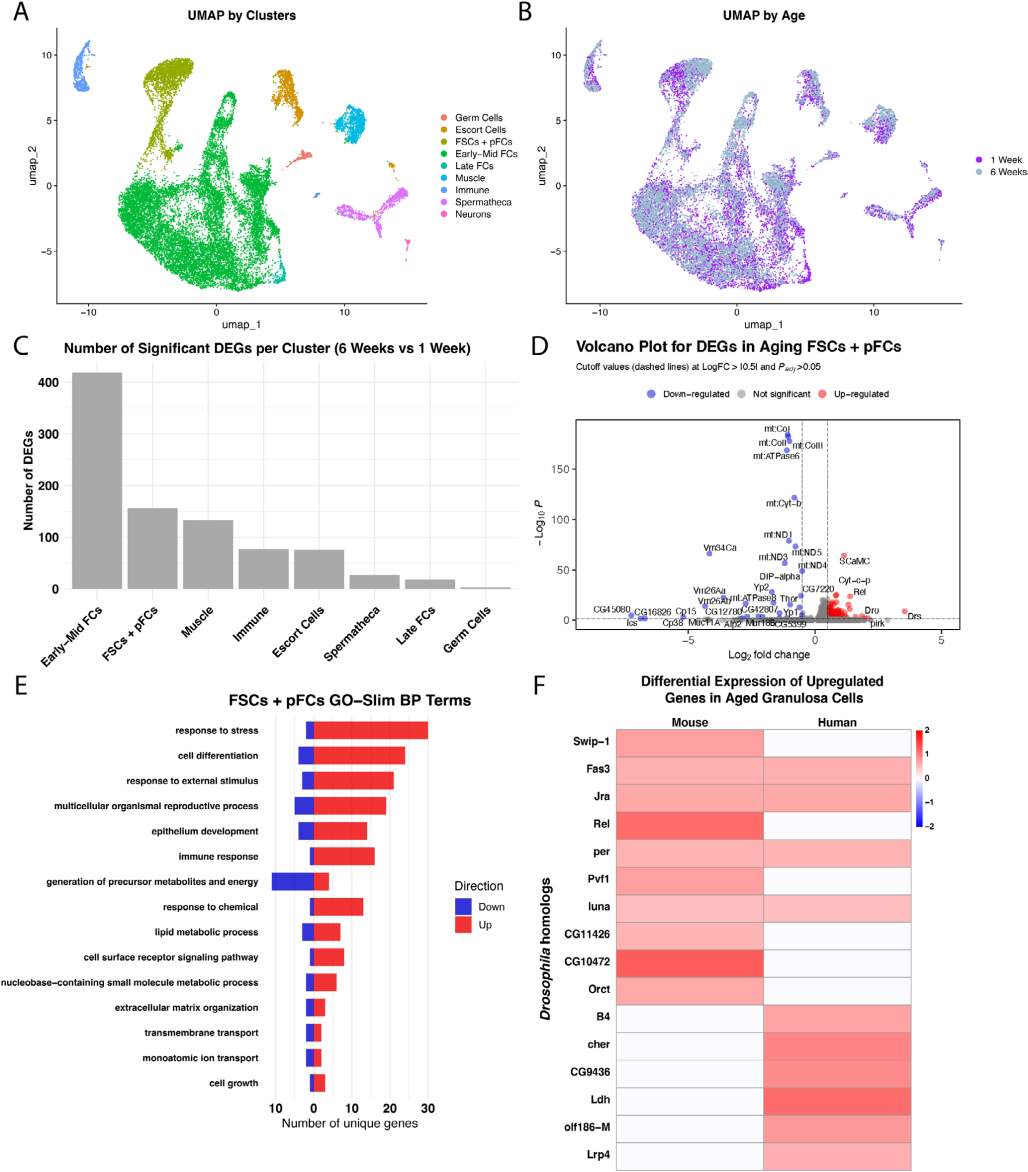
Single‐cell sequencing of young and aged *Drosophila* ovaries reveals large transcriptional differences in aged follicle cells. (A) UMAP of the merged dataset, annotated by the 9 clusters. (B) UMAP of the merged dataset, annotated by the 1‐ and 6‐week old datasets. (C) Ranking of the number of significant differentially expressed genes (DEGs) in each cluster, comparing the 6‐week to the 1‐week old datasets. (D) Volcano plot of DEGs from the FSCs + pFCs cluster. Blue dots are down‐regulated genes, gray dots are not significant genes, and red dots are up‐regulated genes. Significance is indicated by an absolute value log fold‐change greater than 0.5 and adjusted *p*‐value greater than 0.05. (E) Gene ontology terms, using GO‐Slim for the FSCs + pFCs cluster. (F) Heatmap of fly genes with mammalian homologs that are up‐regulated with age, from Gaylord et al. (2025).

To build a comprehensive understanding of the transcriptional differences between young and aged germaria, we performed single‐cell RNA sequencing (scRNA‐seq) of 1‐ and 6‐week‐old ovaries using 10× Genomics system and lllumina sequencing. This produced transcriptional data for 20,818 cells across two timepoints (Table S1) after applying standard quality control filters (Figure S4A,B). Using the standard Seurat workflow, we clustered the cells into nine broad clusters (Figure 4A): Germ cells (*vas*+), Escort cells (*Wnt4*+, *Wnt6*+), FSCs + pFCs (*zfh1*+, *cas*+, *eya*+, *Fas3*+), Early‐Mid FCs (*eya*+, *br*+, *Fas3*+, *mid*+), Late FCs (*br*+, *mid*+, *Yp1*+), Muscle (*Mhc*+), Immune (*atilla*+, *nec*+), and Spermatheca (*lz*+), and Neurons (*pros*+, *nrv3*+) (Figure S5A) (Rust and Nystul 2020; Sun and Spradling 2013; Honti et al. 2009; Robertson et al. 2003; Palmateer et al. 2023; Roy et al. 2013; Wang and Page‐McCaw 2018). We mapped the cells from aged ovaries onto the young ovary dataset using the Seurat integration features (Figure 4B). Mean silhouette scores revealed that most ovarian cell types maintained strong transcriptional separability across age, indicating strong clustering identities (Figure S5B). Additionally, the percent of cells in each cluster was similar between the young and aged datasets (Table S1). Notably, we found that the Early‐Mid FCs have the highest number of differentially expressed genes, followed by FSCs + pFCs and muscle, with germ cells ranking eighth (Figure 4C). In looking at the whole aged ovary, we found 321 differentially expressed genes (DEGs) between the young and aged condition, and 156 DEGs specifically in the FSCs + pFCs cluster (Figure 4D, Figure S5C, Tables S4–, S5). To determine how these transcriptional changes relate to the transcriptional changes in mammalian granulosa cells with age, we merged the FSCs + pFCs cluster with the Early‐Mid FCs cluster to create a more “granulosa‐like” cell cluster, identified the mammalian orthologs of the DEGs in this merged cluster, and cross‐referenced them with a list of age‐associated DEGs from a recently published study of mouse and human aged granulosa cells (Gaylord et al. 2025). Interestingly, we found several genes that are significantly upregulated in both mouse and human granulosa cells that have homologous genes that are significantly upregulated in our aged *Drosophila* “granulosa‐like” cell cluster (Figure 4F). This list includes genes involved in signal transduction, such as the PDGF‐related factor *Pvf1*, and the JNK‐stress pathway effector *Jun‐related antigen*; and immune response genes such as the NFKB1 homolog, *Relish*, and an uncharacterized serine hydrolase, CG10472, that is homologous to mammalian CTRB1 and is predicted to be involved in innate immunity.

### 3.5 Overexpression of *Atg8a* in Early Follicle Cells Prevents Age‐Related Follicle Cell Decline and Rescues Reproductive Capacity

To identify genetic modifications that could counteract the age‐associated decline in fertility, we screened through well‐known genes associated with classic hallmarks of aging, such as cell differentiation, response to stress, and epithelium development (Figure 4E, Figure S5D). Interestingly, we noticed that overexpression of Atg8a::mCherry‐GFP specifically in the early FSC lineage using *109‐30‐Gal4* produced particularly healthy flies. *Atg8a* is an autophagy gene that is essential for the initiation of an autophagosome and fusion of the autophagosome to a lysosome (Xie et al. 2008; Barth et al. 2011; Bali and Shravage 2017) and while it did not come up as a significant DEG in our single‐cell RNA sequencing dataset (Figure S5E), autophagy defects are highly associated with age‐associated defects. We therefore investigated whether upregulating *Atg8a* in follicle cells could improve the age‐related decline in ovarian function.

**FIGURE 5:**
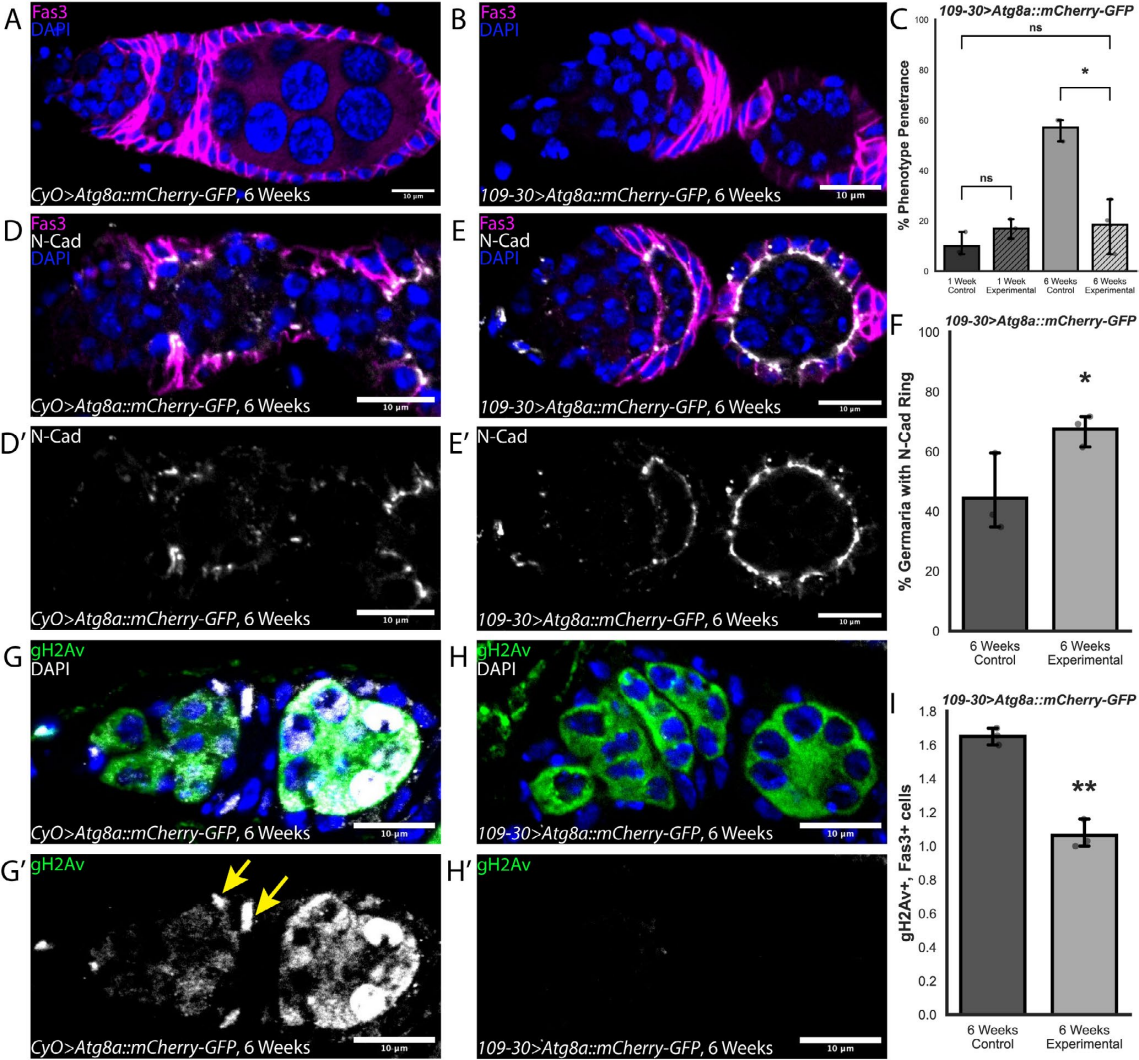
Overexpression of *Atg8a* in follicle cells prevents age‐related follicle cell decline. (A) Germarium from a;CyO;>;Atg8a::mCherry‐GFP; control fly at 6 weeks old stained for Fas3 (magenta) and DAPI (blue). (B) Germarium from a;109‐30;>;Atg8a::mCherry‐GFP; experimental fly stained for Fas3 (magenta) and DAPI (blue). (C) Quantification of percent of germaria with follicle cell phenotypes in 1‐ and 6‐week‐old ;CyO;>;Atg8a::mCherry‐GFP; and ;109‐30;>;Atg8a::mCherry‐GFP;. *n* = 115 and 235 germaria for 1‐week‐old ;CyO;>;Atg8a::mCherry‐GFP; and ;109‐30;>;Atg8a::mCherry‐GFP;, and *n* = 61 and 140 for 6‐weeks‐old ;CyO;>;Atg8a::mCherry‐GFP; and ;109‐30;>;Atg8a::mCherry‐GFP;. (D) Germarium from a 6‐week‐old ;CyO;>;Atg8a::mCherry‐GFP; fly, stained for N‐cad (gray), Fas3 (magenta), and DAPI (blue). (D′) Loss of N‐cadherin ring around developing germ‐cell cysts in 6‐weeks‐old ;CyO;>;Atg8a::mCherry‐GFP;. (E) Germarium from a 6‐week‐old;109‐30;>;Atg8a::mCherry‐GFP; fly, stained for N‐cad (gray), Fas3 (magenta), and DAPI (blue). (E′) Rescue of N‐cad ring around developing germ‐cell cysts in 6‐week‐old ;109‐30;>;Atg8a::mCherry‐GFP; germaria. (F) Quantification of the percent of germaria with the N‐cad ring around germ‐cell cysts in 6‐week‐old ;CyO;>;Atg8a::mCherry‐GFP; and ;109‐30;>;Atg8a::mCherry‐GFP;. *n* = 101 and 118 for 6‐week‐old ;CyO;>;Atg8a::mCherry‐GFP; and ;109‐30;>;Atg8a::mCherry‐GFP;. (G) Germarium from a 6‐week‐old ;CyO;>;Atg8a::mCherry‐GFP; control fly stained for gH2Av (gray), Fas3 (magenta), and DAPI (blue). (H) Germarium from a 6‐week‐old ;109‐30;>;Atg8a::mCherry‐GFP; control fly stained for gH2Av (gray), Fas3 (magenta), and DAPI (blue). (G′, H′) gH2Av identifies sites of DNA damage. (I) Quantification of the number of highly positive gH2Av+, Fas3+ cells per germarium in 6‐week‐old ;CyO;>;Atg8a::mCherry‐GFP; and ;109‐30;>;Atg8a::mCherry‐GFP;. *n* = 77 and 84 for 6‐week‐old ;CyO;>;Atg8a::mCherry‐GFP; and;109‐30;>;Atg8a::mCherry‐GFP;. Scale bars are 10 μm. ns = not significant, *p < 0.05, **p < 0.01, using Welch's *t*‐test (C), Student's *t*‐test (F), and Poisson Dispersion Test (I).

**FIGURE 6:**
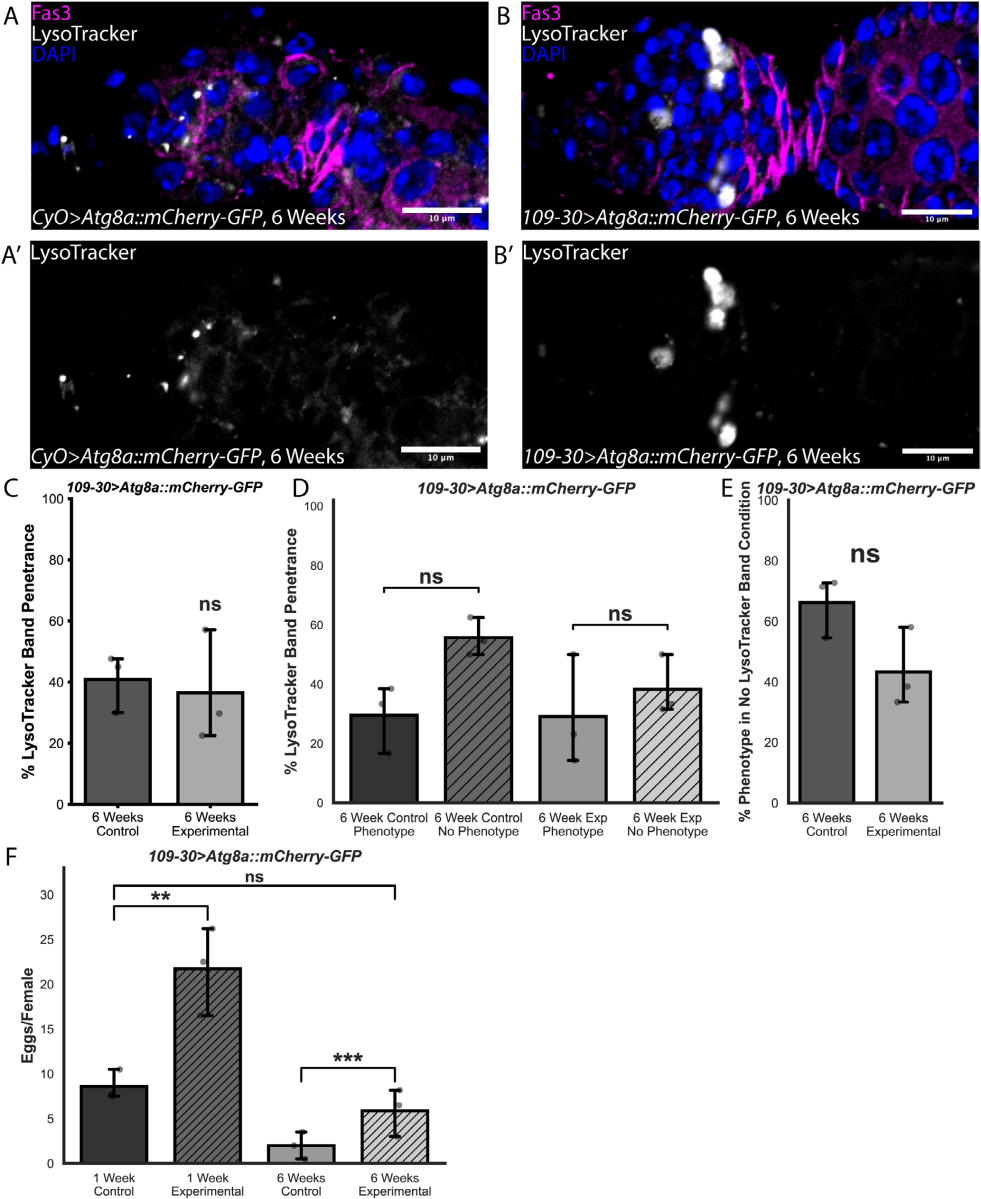
Overexpression of *Atg8a* in follicle cells has a non‐autonomous effect on germ‐cell cyst regulation and rescues egg laying. (A) Germarium from a 6‐week‐old ;CyO;>;Atg8a::mCherry‐GFP; control fly, stained for Lysotracker (gray), Fas3 (magenta), and DAPI (blue). (A′) A Lysotracker‐positive band may be absent anterior to the Region 2a/2b border in phenotype‐positive germaria. (B) Germarium from a 6‐week‐old ;109‐30;>;Atg8a::mCherry‐GFP; experimental fly, stained for Lysotracker (gray), Fas3 (magenta), and DAPI (blue). (B′) Lysotracker‐positive bands may be present anterior to the Region 2a/2b border in phenotype‐negative germaria. (C) Quantification of the percent of germaria with the Lysotracker‐positive band in 6‐week‐old ;CyO;>;Atg8a::mCherry‐GFP; and ;109‐30;>;Atg8a::mCherry‐GFP;. *n* = 51 and 84 germaria for ;CyO;>;Atg8a::mCherry‐GFP; and ;109‐30;>;Atg8a::mCherry‐GFP. (D) Quantification of the percent of 6‐week‐old ;CyO;>;Atg8a::mCherry‐GFP; germaria and percent of 6‐week‐old ;109‐30;>;Atg8a::mCherry‐GFP; with the Lysotracker‐positive band in phenotype‐positive and phenotype‐negative germaria. *n* = 28 ;CyO;>;Atg8a::mCherry‐GFP; phenotype‐positive germaria, *n* = 23 ;CyO;>;Atg8a::mCherry‐GFP; phenotype‐negative germaria, *n* = 36 ;10,930;>;Atg8a::mCherry‐GFP; phenotype‐positive germaria, *n* = 49 ;109‐30;>;Atg8a::mCherry‐GFP; phenotype‐negative germaria. (E) Quantification of phenotype penetrance in germaria where no Lysotracker‐positive band is present for ;CyO;>;Atg8a::mCherry‐GFP; and ;109‐30;>;Atg8a::mCherry‐GFP;. *n* = 29 and 60 germaria for ;CyO;>;Atg8a::mCherry‐GFP; and ;109‐30;>;Atg8a::mCherry‐GFP;. (F) Quantification of eggs laid per female in a 24‐h period for 1‐ and 6‐week‐old ;CyO;>;Atg8a::mCherry‐GFP; and ;109‐30;>;Atg8a::mCherry‐GFP; flies. *n* = 10 and 11 vials for 1‐week‐old ;CyO;>;Atg8a::mCherry‐GFP; and ;109‐30;>;Atg8a::mCherry‐GFP;, and *n* = 5 and 8 vials for 6‐week‐old ;CyO;>;Atg8a::mCherry‐GFP; and ;109‐30;>;Atg8a::mCherry‐GFP;. Scale bars are 10 μm. ns = not significant, **p < 0.01, ***p < 0.001, using Welch's *t*‐test (C, E), Binomial General Linearized Mixed Model (D), and Poisson Distribution Test (F).

Indeed, we found that there was a significant reduction in the frequency of follicle cell phenotypes relative to an internal control in aged ovaries (Figure 5A–C). Interestingly, we observed a slight increase in follicle cell phenotype frequency in young ovaries (Figure 5C), suggesting that at least some of the beneficial effects of *Atg8a* overexpression may be specific to the aged condition. In addition, we observed a significant increase in the percentage of aged germaria that have maintained the ring of N‐cad upon overexpression of Atg8a::mCherry‐GFP compared to control (Figure 1G′,H′). Moreover, we found that overexpression of Atg8a::mCherry‐GFP decreased the number of highly expressing gH2Av positive follicle cells (Figure 1G,H,G′,H′,I). Additionally, we found that overexpression of Atg8::mCherry‐GFP in follicle cells has cell non‐autonomous effects on the aged germline. While overexpression of Atg8::mCherry‐GFP did not significantly alter the frequency of germaria with LysoTracker^+^ germ‐cell cysts in Region 2a, it eliminated the association between the presence of follicle cell phenotypes and the lack of a LysoTracker^+^ cyst in Region 2a (Figure 6A–D). Finally, we assayed for egg‐laying to determine if overexpression of *Atg8a* in follicle cells could rescue age‐related decline in reproductive capacity (Partridge et al. 1986). We found that egg‐laying increased from 2 ± 1.5 eggs per female per day in aged control *Drosophila* to 5.9 ± 2.6 eggs per female per day in *Atg8a* overexpression condition, a nearly 2‐fold increase in egg‐laying (Figure 6F). Importantly, egg‐laying in aged *Atg8a* overexpression condition was not significantly different from that of young control *Drosophila* (Figure 6F). Taken together, these data indicate that overexpression of *Atg8a* improves age‐related follicle cell decline in tissue integrity, cell adhesion, DNA damage repair, and reproductive capacity in aged ovaries.

## 4 Discussion

Here, we have identified several key genetic and cellular changes that accumulate with age in the *Drosophila* follicle epithelium and discovered a targeted intervention, overexpression of *Atg8a* specifically in the early FSC lineage, that ameliorates these phenotypes. Several of the defects we identified, such as gaps in the follicle epithelium and the lack of an interfollicular stalk, are incompatible with follicle development and thus likely to be significant causes of reduced fecundity in aged flies. In addition, we identified intracellular phenotypes, including increased DNA damage and a disruption of cellular junctions, that indicate a loss of normal follicle cell function. Moreover, our scRNA‐seq data provide a comprehensive account of cell type specific changes in gene expression with age. Interestingly, our analysis predicts that there are more differentially expressed genes in the follicle lineage than any other cell type in the ovary. Additionally, several of the highly upregulated genes in mammalian aged granulosa cells are homologous to significantly upregulated genes in the aged *Drosophila* follicle lineage. Taken together, these observations emphasize the importance of the early FSC lineage in *Drosophila* ovarian aging and provide a baseline description of age‐associated changes in this tissue that can be used to evaluate the effects of experimental interventions that target follicle cells. Notably, the follicle cell phenotypes we observed, including DNA damage and cell cycle alterations, are also hallmarks of reproductive aging in mammals (Balough et al. 2024), highlighting the opportunity to understand conserved mechanisms of reproductive aging through these studies.

We were surprised to find that overexpression of *Atg8a* with *190‐30‐Gal4* is sufficient to increase fecundity in both young and old flies. *109‐30‐Gal4* expression is restricted to a relatively small population of somatic cells located in the posterior half of the germarium and the first 2–3 budded follicles, yet genetic manipulation of this small population increased the overall output of the whole ovary. This demonstrates that oogenesis is not operating at maximum capacity under the growth conditions we used, and that the function of these cells in the early FSC lineage is rate‐limiting. In addition, though fecundity still declines with age in flies with *Atg8a* overexpression, it is notable that the rate of egg laying in the aged condition is not significantly different from that of young control flies. Thus, despite the impact of other sources of decline that inevitably occur with age, these flies still maintain a youthful level of fecundity in the aged condition. These findings are consistent with emerging evidence that targeting the effects of ovarian somatic cell aging in mammals may slow reproductive aging. For example, the pro‐inflammatory signal, CD38, which is primarily expressed in endothelial and immune cells, increases with age and global inhibition of this increase enhances fertility in aged mice (Yang et al. 2024). Likewise, interventions that reduce fibrosis caused by ovarian somatic cells are being considered as possible therapeutic avenues (Umehara et al. 2022). An advantage of these interventions is that, by focusing on the support cells, they avoid direct manipulation of the germ cells that will contribute to the next generation. The genetic tools available here enabled us to use targeted genetic manipulations to study the role of somatic cells in reproductive aging.

The cells of the early FSC lineage may influence the rate of oogenesis through their direct role in facilitating follicle formation as well as through signaling with germ cells that helps coordinate the supply of follicle cells with demand from newly‐produced germ‐cell cysts. A key factor in this coordination process is the nutrition‐dependent checkpoint at Region 2a. This checkpoint and a second one that occurs in mid oogenesis may be orthologous to a process in the mammalian ovary in which granulosa cells secrete signals to induce follicle atresia (Pritchett et al. 2009). We found that the frequency of germaria with a dying cyst in Region 2a did not change between young and old flies or even with the overexpression of *Atg8*. However, the presence of age‐associated follicle cell phenotypes is linked to the absence of a dying cyst in Region 2a, raising the interesting possibility that germaria without a recent activation of this checkpoint are more susceptible to a loss of coordination between germ cells and follicle cells. Alternatively, germaria with follicle cell phenotypes may be less likely to activate the checkpoint, possibly leading to further loss of coordination. Understanding how a loss of coordination at this stage of oogenesis contributes to overall fecundity rates will be an important topic for future studies.

Mechanistically, *Atg8a*, which is homologous to mammalian LC3, is well‐studied as a key regulator of autophagy (Kabeya et al. 2000; Gatica et al. 2018). Autophagy is a protective cellular function that promotes the degradation of proteins, organelles, and other cellular components to mitigate the accumulation of damaged biomolecules over time (Gatica et al. 2018). Autophagy is regulated by a cascade of signals, with several ATG (autophagy‐related genes) involved (Gatica et al. 2018; Bali and Shravage 2017). *Atg8a* resides on the membrane of autophagosomes during formation and expansion and is known to increase in expression in response to stress conditions such as starvation and reactive oxygen species accumulation in the *Drosophila* ovary (Xie et al. 2008; Barth et al. 2011; Bali and Shravage 2017). In addition, an upstream regulator, *Atg7*, is required in follicle cells for coordination between germ cells and follicle cells in the progression of oogenesis (Barth et al. 2011). In the mammalian ovary, LC3 expression and standard indicators of autophagy activity, such as lysosome number and autolysosome fusion, decline with age, thus reducing the capacity of this protective mechanism (Peters et al. 2021; Yao et al. 2024). Autophagy is critical for DNA damage mediation, where autophagy is promoted by the DNA damage response and repair pathways (Czarny et al. 2015; Gomes et al. 2017). Additionally, autophagy, and *Atg8a* specifically, also works upstream to prevent DNA damage and oxidative stress (Nagy et al. 2018; Simonsen et al. 2008). Consistent with this, we found that overexpression of *Atg8a* decreases the accumulation of DNA damage in follicle cells. Likewise, autophagy machinery is known to traffic E‐cadherin to lysosomes (Damiano et al. 2020). This suggests that similar mechanisms may facilitate the maintenance of proper N‐cad localization in ovarioles with *Atg8a* overexpression, as we have shown. These anti‐aging effects at the cellular level likely contribute to the improved function of the follicle epithelium at the tissue level.

In summary, this study contributes to a growing body of literature that highlights the importance of ovarian somatic cells in reproductive health and aging. Further studies into other features of follicle cell aging and how they contribute to the decline in overall ovarian function will be of interest. In addition, with emerging evidence that ovarian health contributes to overall organism health, our findings will support future efforts to investigate the effects of follicle cell health on other organ systems and lifespan.

## Author Contributions

E.A.W. and T.G.N. contributed to conceptualization, data curation, formal analysis, funding acquisition, supervision, and visualization. E.A.W. contributed to investigation, and writing – original draft preparation. T.G.N. contributed to methodology, project administration, and writing – review and editing.

## Funding

This work was supported by the National Institute of General Medical Sciences of the National Institutes of Health under Award Number R35GM136348 to T.G.N., a Bakar Aging Research Institute Investigator Award to T.G.N., the Eunice Kennedy Shriver National Institute Of Child Health & Human Development of the National Institutes of Health under Award Number T32HD007470, and a National Science Foundation Graduate Research Fellowship under Grant No 2445150 to E.A.W. CAT is supported by UCSF PBBR, RRP IMIA, and NIH 1S10OD028511‐01 grants.

## Ethics Statement

This manuscript is not currently under consideration for publication elsewhere and does not involve the use of human subjects or vertebrate animal models. The authors do not have any financial conflicts of interest to declare.

## Conflicts of Interest

The authors declare no conflicts of interest.

## Supporting information


**Figure S1:** Schematics of aging phenotypes for characterizations. (A) Schematic of no phenotype condition. (B) Schematic of epithelial gapping phenotype, where germ cysts are not fully encapsulated by early follicle cells. (C) Schematic of dysregulated epithelium phenotype, where early follicle cells are not forming a single‐layer epithelium. (D) Schematic of epithelial cell death phenotype, where large groups of follicle cells appear rounded, rather than cuboidal. (E) Schematic of an example of impaired stalk development phenotype, where stalk cells may not be present between developing follicles.

**Figure S2:** Further characteristics of the aging ovary. (A, B) Germarium from a 1‐week‐old (A) and 6‐week‐old (B) *w1118(x);;* flies, stained for Vkg (green), Fas3 (magenta), and DAPI (blue). (A′, B′) Vkg encodes for a subunit of collagen IV, which integrates into the basement membrane around the perimeter of the germarium. (C) Quantification of the average intensity of Vkg around the perimeter of the germarium at 1‐ and 6‐week‐old. *n* = 26 and 27 germaria for 1‐ and 6‐week‐old. (D, E) Germarium from a 1‐week‐old (C) and 6‐week‐old (D) *w1118(x);;* flies, stained for TUNEL (green), Fas3 (magenta), and DAPI (blue). (D′, E′) TUNEL stains sites of DNA fragmentation, indicating final stages of apoptosis. (F) Quantification of the average number of TUNEL‐positive, Fas3‐positive cells per germarium at 1‐ and 6‐week‐old. *n* = 110 and 119 germaria for 1‐ and 6‐week‐old. Scale bars are 10 μm. ns = not significant, using Welch's *t*‐test (C) and Poisson Distribution Test (F).

**Figure S3:** Large LysoTracker‐positive structures in Region 2a are not canonical lysosomes or late endosomes. (A) Germarium from a 1‐week‐old*;;Lamp1::3x‐mCherry* fly, stained for RFP (magenta) and DAPI (blue). (A′) Lamp1 is a membrane protein on lysosomes and does not have the same pattern as the large Lysotracker‐positive structures seen in Region 2a. (B) Germarium from a 1‐week‐old*;;w1118(x)* fly, stained for Rab7 (green), Lysotracker (gray), and DAPI (blue). (B′) Rab7 is a protein associated with late endosomes and does not colocalize with the large Lysotracker‐positive structures seen in Region 2a.

**Figure S4:** Quality control plots for single‐cell RNA sequencing of young and aged ovaries. (A, B) Violin plots for the number of RNA features by age and replicate. (A′, B′) Violin plots for the number of RNA counts by age and replicate. (A″, B″) Violin plots for the percent mitochondria reads per cell ID by age and replicate.

**Figure S5:** Cluster identification and additional analysis of single‐cell RNA sequencing of young and aged ovaries. (A) Dot plot of gene expression for markers associated with each cluster of cells. (B) Mean silhouette score for each cluster compared against its own dataset for 1‐ and 6‐week‐old. (C) Volcano plot of DEGs for the whole ovary. Blue dots are down‐regulated genes, gray dots are not significant genes, and red dots are up‐regulated genes. Significance is indicated by an absolute value log fold‐change greater than 0.5 and adjusted *p*‐value greater than 0.05. (D) Gene ontology terms, using GO‐Slim for the whole ovary. (E) Dot plot of gene expression for *Atg8a* in each cluster for 1‐ and 6‐week‐old.

**Table S1:** Cell counts by dataset.

**Table S2:** Top 50 DEGs by cluster comparing the 1‐ and 6‐week‐old datasets.

**Table S3:** Number of significant DEGs by cluster comparing the datasets from 1 and 6 weeks old.

**Table S4:** List of the 321 significant DEGs for whole aging ovary.

**Table S5:** List of the 156 significant DEGs for whole aging ovary.

## Data Availability

Raw data files, code for generating graphs and statistical analysis, and code for building the Seurat object are available via the GitHub repository (https://github.com/NystulLab/WolfgramAgingOvary.git). FASTQ are available on GEO (accession # GSE318563).
