# Supplementary figures and images for: A Decline in Follicle Cell Function Is a Major Driver of Drosophila Ovarian Aging
Source: Aging Cell. 2026 Apr 30;25(5):e70529. doi: 10.1111/acel.70529 (PMC13132801; doi:10.1111/acel.70529)

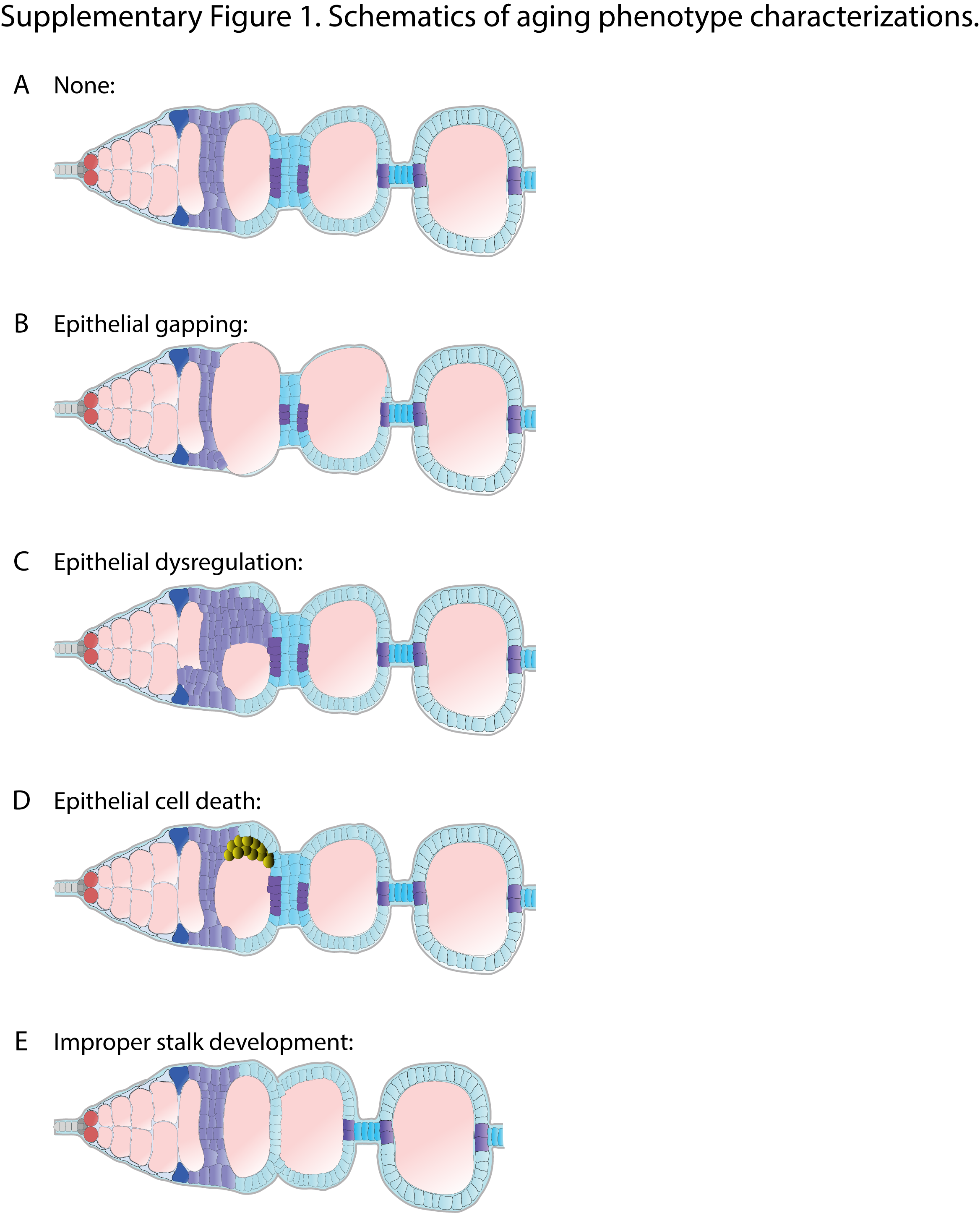

Supplement: Supplementary file 1 — Figure S1: Schematics of aging phenotypes for characterizations. (A) Schematic of no phenotype condition. (B) Schematic of epithelial gapping phenotype, where germ cysts are not fully encapsulated by early follicle cells. (C) Schematic of dysregulated epithelium phenotype, where early follicle cells are not forming a single‐layer epithelium. (D) Schematic of epithelial cell death phenotype, where large groups of follicle cells appear rounded, rather than cuboidal. (E) Schematic of an example of impaired stalk development phenotype, where stalk cells may not be present between developing follicles. [file ACEL-25-e70529-s007.png]

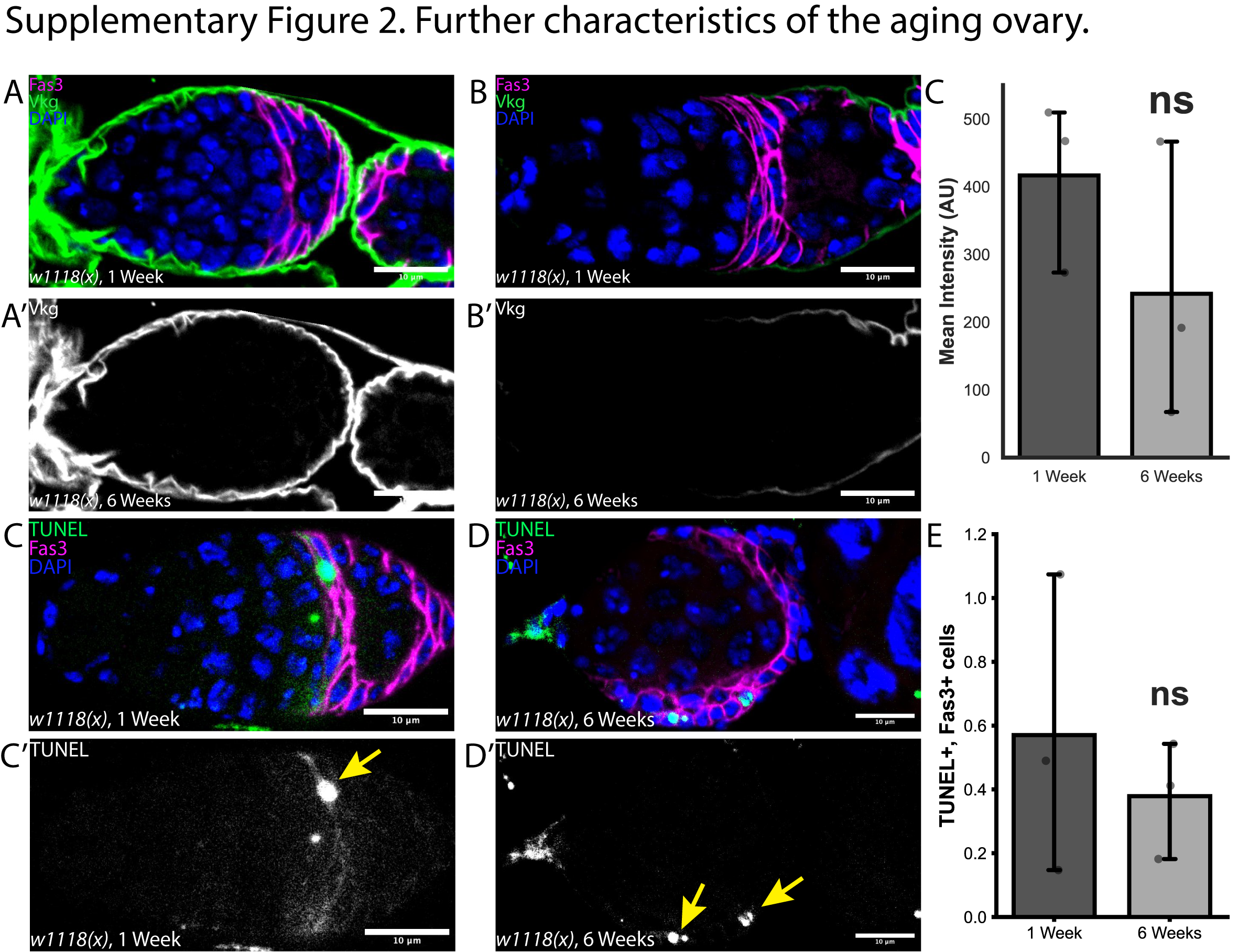

Supplement: Supplementary file 2 — Figure S2: Further characteristics of the aging ovary. (A, B) Germarium from a 1‐week‐old (A) and 6‐week‐old (B) w1118(x);; flies, stained for Vkg (green), Fas3 (magenta), and DAPI (blue). (A′, B′) Vkg encodes for a subunit of collagen IV, which integrates into the basement membrane around the perimeter of the germarium. (C) Quantification of the average intensity of Vkg around the perimeter of the germarium at 1‐ and 6‐week‐old. n = 26 and 27 germaria for 1‐ and 6‐week‐old. (D, E) Germarium from a 1‐week‐old (C) and 6‐week‐old (D) w1118(x);; flies, stained for TUNEL (green), Fas3 (magenta), and DAPI (blue). (D′, E′) TUNEL stains sites of DNA fragmentation, indicating final stages of apoptosis. (F) Quantification of the average number of TUNEL‐positive, Fas3‐positive cells per germarium at 1‐ and 6‐week‐old. n = 110 and 119 germaria for 1‐ and 6‐week‐old. Scale bars are 10 μm. ns = not significant, using Welch's t‐test (C) and Poisson Distribution Test (F). [file ACEL-25-e70529-s004.png]

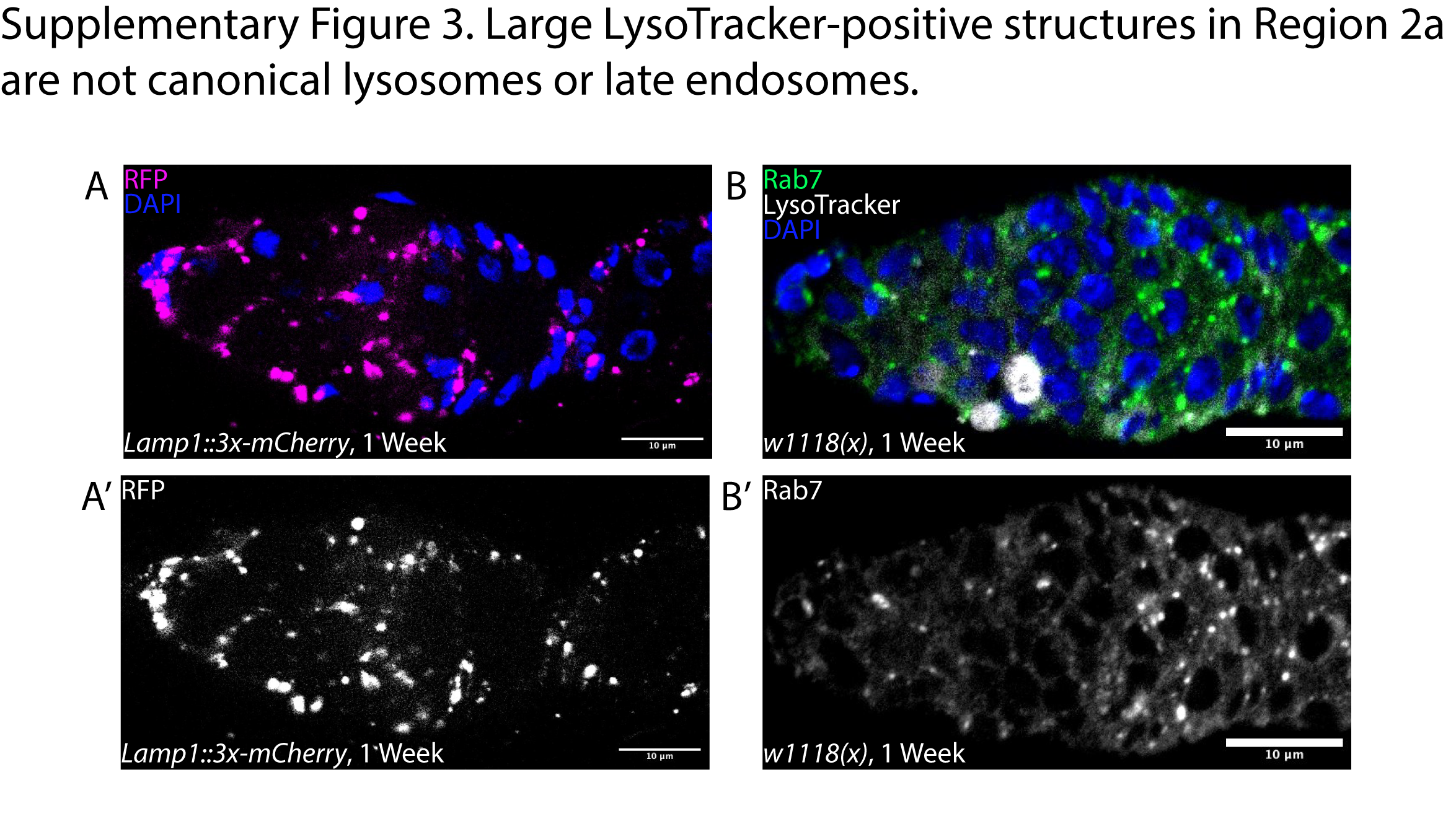

Supplement: Supplementary file 3 — Figure S3: Large LysoTracker‐positive structures in Region 2a are not canonical lysosomes or late endosomes. (A) Germarium from a 1‐week‐old;;Lamp1::3x‐mCherry fly, stained for RFP (magenta) and DAPI (blue). (A′) Lamp1 is a membrane protein on lysosomes and does not have the same pattern as the large Lysotracker‐positive structures seen in Region 2a. (B) Germarium from a 1‐week‐old;;w1118(x) fly, stained for Rab7 (green), Lysotracker (gray), and DAPI (blue). (B′) Rab7 is a protein associated with late endosomes and does not colocalize with the large Lysotracker‐positive structures seen in Region 2a. [file ACEL-25-e70529-s002.png]

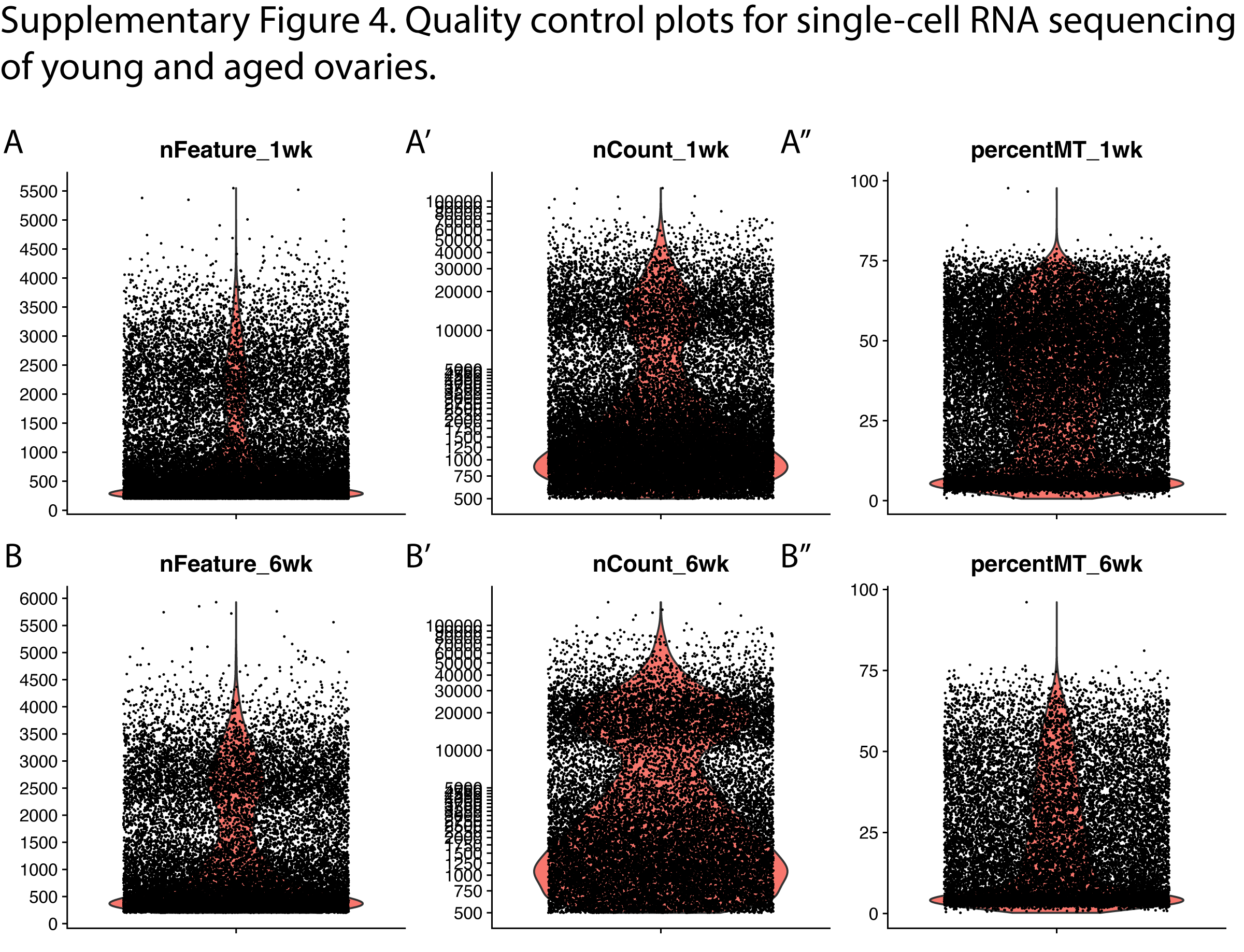

Supplement: Supplementary file 4 — Figure S4: Quality control plots for single‐cell RNA sequencing of young and aged ovaries. (A, B) Violin plots for the number of RNA features by age and replicate. (A′, B′) Violin plots for the number of RNA counts by age and replicate. (A″, B″) Violin plots for the percent mitochondria reads per cell ID by age and replicate. [file ACEL-25-e70529-s009.png]

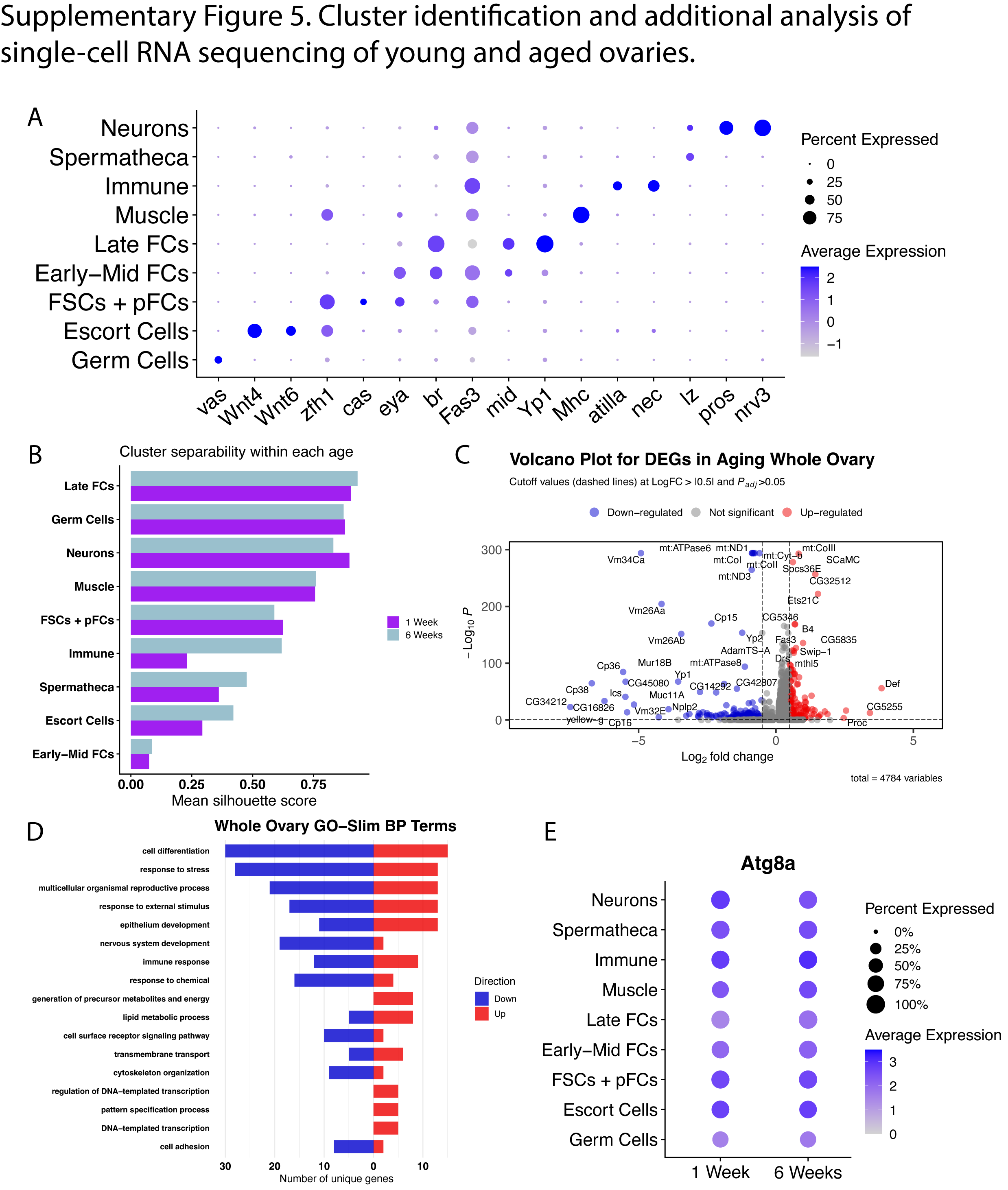

Supplement: Supplementary file 5 — Figure S5: Cluster identification and additional analysis of single‐cell RNA sequencing of young and aged ovaries. (A) Dot plot of gene expression for markers associated with each cluster of cells. (B) Mean silhouette score for each cluster compared against its own dataset for 1‐ and 6‐week‐old. (C) Volcano plot of DEGs for the whole ovary. Blue dots are down‐regulated genes, gray dots are not significant genes, and red dots are up‐regulated genes. Significance is indicated by an absolute value log fold‐change greater than 0.5 and adjusted p‐value greater than 0.05. (D) Gene ontology terms, using GO‐Slim for the whole ovary. (E) Dot plot of gene expression for Atg8a in each cluster for 1‐ and 6‐week‐old. [file ACEL-25-e70529-s001.png]
